# Quantitative pinch stimulator for exploring evoked nociceptive responses: A pilot study

**DOI:** 10.1186/1475-925X-9-79

**Published:** 2010-11-24

**Authors:** Chih-Ping Chen, Wen-Li Liao, Yi-Li Tseng, Pen-Li Lu, Yu-Chun Lo, You-Yin Chen, Fu-Shan Jaw

**Affiliations:** 1 Institute of Biomedical Engineering, College of Engineering and College of Medicine, National Taiwan University, Taipei, Taiwan; 2 Institute/Department of Biomedical Engineering, National Yang-Ming University, Taipei, Taiwan

## Abstract

**Background:**

A mechanical noxious stimulator is useful for studies of pain, both for clinic and basic research. We propose to use a pinch stimulator that can not only generate a quantitative, reproducible noxious pinch but also simultaneously provide a synchronous external trigger signal, which is essential for acquisition of evoked potentials.

**Methods:**

For ethical considerations, audible and visual aids were incorporated so that pinch force could be regulated within a predetermined level. Reproducibility of the nociceptive responses evoked by this device was validated. The device was constructed with a simple circuit, and the element build-in was delicately selected for the minimum required to produce evoked potentials.

**Results:**

The magnitude of the force output is linearly proportional to the volts produced by the device (i.e., during the pinch). Increases in force correspond to increases in the number of action potentials induced.

**Conclusions:**

This device may be useful for studying the mechanisms of nociceptive signal processing in the brain through application of reproducible, noxious pinch stimuli.

## Background

Nociception is a primitive sense that plays an important role in protecting organisms from dangerous conditions. However, many researchers have encountered difficulties in studying nociception, due to lacking of appropriate noxious stimulators that can induce a reproducible pain sensation. Electrical stimulators are commonly used [1,2] because they are easy to synchronize. However, electrical stimulation will activate various types of nerve fibers, which results in ambiguous sensation. CO_2 _laser has been used as specifically noxious heat stimulation [3]. However, nociceptive responses induced are not the same as the responses evoked by mechanical pinch. Noxious pinch stimulation is valuable and easy to apply.

Calibrated forceps for inducing pain sensations have been commercially available [4-6]. However, these forceps usually lack a synchronous output to trigger an analogue-to-digital (A/D) card or an oscilloscope for response acquisition. They are also lacking an alarm to avoid damaging the tissue by applying excessive forces.

Based on these considerations, we propose a mechanical, noxious pinch stimulator that can not only simultaneously generate a trigger signal for data acquisition but also provide quantitative noxious stimuli within a predetermined level. The former feature is especially important for studies of nociception utilizing event-related evoked responses. The latter feature, use of an acoustic signal that determines the noxious pinch force by users, prevents tissue injury by excessive forces and applies a consistent force easily. The proposed pinch stimulator has been utilized for exploring nociceptive responses in the primary somatosensory cortex of rats. The results suggest that this device is suitable for noxious studies, especially for stimulus-triggered recording of neuronal activities.

## Materials and methods

### Principles of the pinch stimulator design

#### Selection of transducer and forceps

The transducer used in the pinch stimulator should be sensitive, small, and have an adequate frequency response. There are two kinds of strain gauges commonly available: one has a resistance of 120 Ω and the other has a resistance of 350 Ω. Because the sensitivity of a strain gauge is proportional to its resistance, a 350-Ω strain gauge was used. Therefore, a strain gauge (FLA-1-350-11-1L, Tokyo Sokki Kenkyujo, Japan) with a dimension of 6 mm^2 ^was selected. The strain gauge was attached one-third of the distance from the tips of the Dumont #2AP forceps (catalog # 1220-21, Fine Science Tools, USA; dimension: 1.5 mm × 0.7 mm). This distance was selected so that strain gauge sensitivity to bending was maintained but was not easily perturbed by the finger of the user. The tips of the selected forceps were blunt in order to avoid extraneous injuries to the tissue during pinching.

#### Circuit design

**Figure 1:**
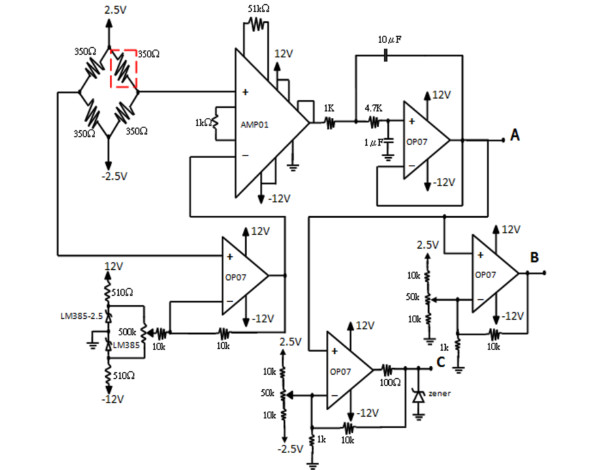
**The complete circuit diagram of the pinch stimulator**. The three outputs include (A) the analog voltage signal that encodes the force of a pinch, (B) an alarm signal, and (C) a TTL-compatible synchronous output.

The complete circuit for the pinch stimulator is shown in Figure 1. Some essential functions contained within the designed circuitry, such as temperature compensation, offset adjustment, and adequate filtering, are described in detail: An instrumentation amplifier (AMP01, Analog Devices, USA) with a gain of 1000, was used to amplify the small signals detected by the strain gauge. A high precision operational amplifier (OP07, Analog Devices, USA) was used to ensure that the DC offset compensation of the strain gauge transducer was calibrated to zero before the amplification to prevent saturation of the output signal. Because the pinch forces were low in frequency, a second-order low-pass Butterworth filter with a corner frequency of 23 Hz was used. Thus, the 60-Hz background noise could be properly filtered, and a high-precision operational amplifier was used to achieve the required stability at low frequencies. A Wheatstone bridge was used for temperature compensation in order to prevent temperature-related drift in the output signal caused by changes in ambient temperature.

### Animal experimentation

#### Preparation

Animal usage was approved by the Institutional Animal Care and Use Committee of National Taiwan University Hospital. Three male Wistar rats weighing 280-350 g were used. All efforts were made to minimize the number of animals used and their suffering. Rats were initially anesthetized with ketamine (50 mg/ml, i.p.) and Rompun (0.3 mg/ml; i.p.), and dilute ketamine (1.5 mg/ml; i.v.) was injected during the experiment using an infusion pump with infusion rate of 1.5 ml/h. Bregma was used as the origin, and the coordinates for recording were derived from the *Atlas of the Rat Brain *[7]. Extracellular action potentials (APs) were recorded in the tail representation area of the primary somatosensory cortex (1.8 to 1.9 mm lateral to the midline, 1.8 to 1.9 mm caudal to bregma, depth of 300 to 1500 μm from the brain surface) [8]. The rectal temperature of the rats was kept at 37°C ± 0.5°C with a homeothermic blanket.

#### Noxious-pinch stimulation

Two output BNC connectors were located on the panel of this stimulator to represent the output voltage transformed from the force input and the external synchronous trigger, respectively. In addition, the knob from a buzzer alarm was installed nearby to set the pain level being administered. After the baseline response level was set to zero, the user applied a series of forces to his or her palm of the hand to determine the level of applied force that would induce a pain sensation. The average force that induced pain sensations was about 91.3 ± 2.0 gw. After the threshold was set, the stimulator emitted an acoustic alarm if the threshold was exceeded in order to prevent excessive and irreversible injury to the skin during pinching. During the skin pinch, a 5-V TTL pulse was generated to trigger the A/D card (PCI-MIO-16E-4, National Instrument, USA) or the oscilloscope for acquisition of the evoked responses. The skin in the middle of the rat tail, which is innervated by the 3^rd ^sacral root (S3), was stimulated by pinches [8]. To begin, the user confirmed that the force of the applied pinch achieved noxious levels. Next, the user confirmed that the stimulus produced a slight withdraw reflex in rats under light anesthesia. Observable skin injury (red or swollen tissue) was prevented as much as possible.

#### Recording and analysis of evoked APs

Neuronal responses evoked by the noxious pinches were recorded through a glass microelectrode filled with 3 M NaCl. Evoked APs were recorded, both in the presence and absence of the noxious pinches. Signals detected were then amplified (× 2500), filtered (60-Hz notch filter and 300-Hz/5-kHz band-pass filter), and saved in a computer with an A/D card. A 64-kHz sampling rate and data length of 6400 points were the parameters used to digitize the acquired signals. Force output was also simultaneously recorded. Evoked APs were analyzed through a spike-sorting program written with MATLAB (R2007a, The Mathworks, USA).

## Results

### Verification of the specifications of the pinch stimulator

**Figure 2:**
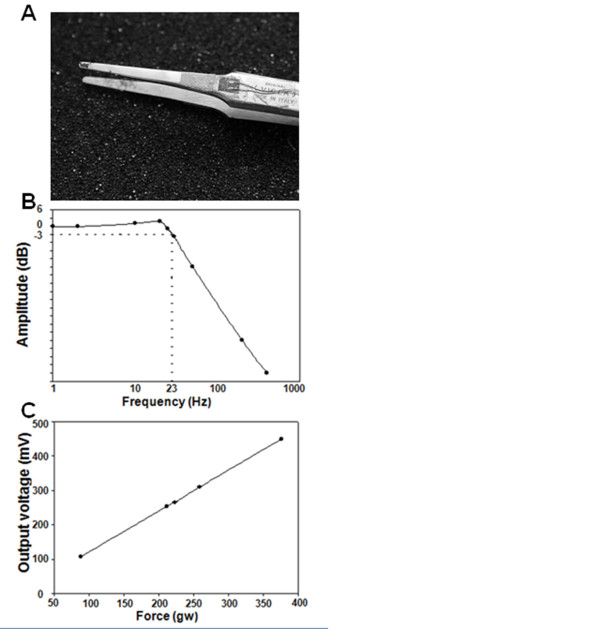
**Specifications of the pinch stimulator**. (A) This photograph shows how the strain gauge attaches to the forceps (Dumont, type 2A). (B) Shown is the frequency response. (C) The transfer function of the stimulator, i.e., the relationship between the output voltage and the force produced by the forceps, is shown.

Figure 2A shows the forceps and the sites of strain gauge attachment. As shown in Figure 2B, the bandwidth of the pinch stimulator was skillfully disposed at 23 Hz in order to authentically present the frequency response transformed from the force output and reduce interference from the 60-Hz noise. For calibration and convenience, a miniature pressure sensor (PS-70KAM260, Kyowa Electronic Instruments, Japan) transformed the force generated by the pinches into the corresponding voltage output, which was a linear relationship, as shown in Figure 2C.

### Evoked nociceptive response from the primary somatosensory cortex of the rat

**Figure 3:**
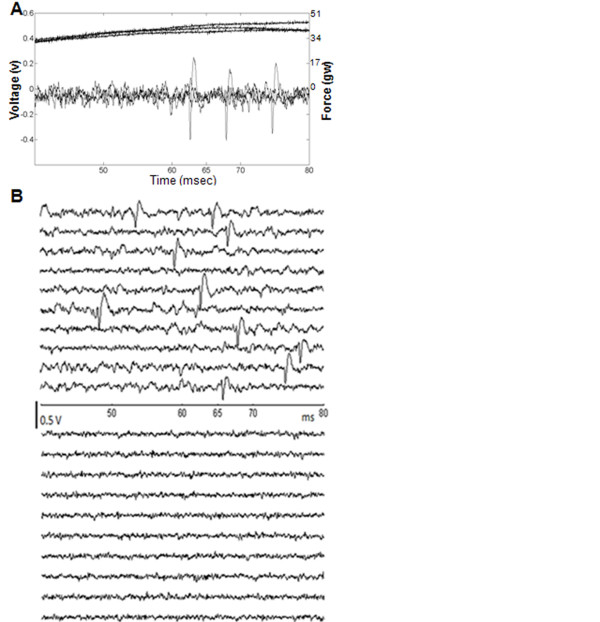
**Noxious-pinch evoked APs (× 2500)**. (A) To show the reproducibility of the applied forces at the pain threshold, three trials are superimposed. The starting point of the upper traces illustrates that the threshold has been reached, and a synchronous output for triggering data acquisition is simultaneously generated, which corresponds to the 0-ms time point on the x-axis. The lower portion of this panel shows the three superimposed evoked responses. (B) Comparison between APs generated by noxious pinch (upper traces) or control conditions (lower traces). The coordinates of the recorded unit was 1.85 mm lateral to the midline and 1.8 mm caudal to bregma, with a depth of 1050 μm. As shown previously [3], the fast nociceptive responses recorded from the primary somatosensory cortex had conduction latencies of approximately 60 ms (A-_δ _fibers). Thus, only the evoked APs that occurred during the 50 to 80 ms after pinch are shown.

As shown in Figure 3A, the upper trace represents the pinch force and the lower trace represents the corresponding evoked APs. In addition, the upper ten cycles shown in Figure 3B represent the APs evoked by the noxious pinch whereas the lower ten cycles were recorded without any pinch stimuli applied to the rat, which was regard as the control trial. The conduction velocity calculated for the distance from the mid-tail to the recording microelectrode (0.3 m) divided by 50-80 ms was 3.75-6.00 m/s, which was within the range of conduction velocity of A-delta fibers.

## Discussion and Conclusions

Compared to other superficial sensory modalities, intensive nociceptive stimulation will cause tissue damage or inflammation, which usually causes allodynia and hyperalgesia, and is generally difficult to reproduce. The proposed pinch stimulator met the requirements mention above and provided an external trigger signal to synchronize the pinch with acquisition of the evoked neuronal responses. Commercially available pinchers that quantify the force output of the pinch do not satisfy certain criteria for studies of pain or nociception, such as studies of evoked responses. With the assistance of a synchronized trigger signal, evoked nociceptive responses can be more easily acquired and analyzed.

The evoked APs induced by the pinch stimulator were verified with a spike-sorting program. During the experiments, with the aid of the audible alarm, the parts of the skin that received the pinch stimuli were not irreversibly injured or characterized by obvious swelling.

In sum, this device provides visual and acoustic aids to prevent application of forces that exceed a given level, preventing tissue injury. Hence, a consistent and quantitative force can be reproducibly applied and read from the waveform on the oscilloscope. In the present report, the performance of the proposed pinch stimulator has been proved from the designed experiments for studies of evoked nociceptive responses. Due to the audible alarm signal, noxious stimuli could be repeatedly applied in order to gather data to validate the signal. In addition, it is valuable for reproducing evoked neuronal activities, intracellular recording, or both. In the present work, the pinch stimulator was designed to overcome several predicaments prevalent in pain studies. To ensure that other laboratories could duplicate this device, the complete circuit and construction of the strain gauge were described in detail. We hope this information is helpful to our colleagues.

## Competing interests

The authors declare that they have no competing interests.

## Authors' contributions

CPC participated in the design of the study, performed the experiments, and drafted and revised the manuscript. WLL and YLT evaluated the experiment and revised the manuscript. PLL, YCL and YYC participated in data analysis and revised the manuscript. FSJ participated in the theoretical aspects of study design, circuit design, and supervised the experiment. All authors read and approved the final draft of the manuscript.
